# Supply-chain factors and antimicrobial stewardship

**DOI:** 10.2471/BLT.22.288650

**Published:** 2023-04-17

**Authors:** Nduta Kamere, Victoria Rutter, Derick Munkombwe, Dorothy Atieno Aywak, Eva Prosper Muro, Felix Kaminyoghe, Kalidi Rajab, Mashood Oluku Lawal, Naomi Muriithi, Ndinda Kusu, Oluwatoyin Karimu, Shuwary Hughric Adekule Barlatt, Winnie Nambatya, Diane Ashiru-Oredope

**Affiliations:** a Commonwealth Pharmacists Association, London, England.; b Department of Pharmacy, University of Zambia, Lusaka, Zambia.; c Division of Pharmaceutical Services, Kenyatta National Hospital, Nairobi, Kenya.; d Kilimanjaro Christian Medical University College, Kilimanjaro, United Republic of Tanzania.; e Pharmaceutical Society of Malawi, Lilongwe, Malawi.; f Makerere University Pharmacy Department, Kampala, Uganda.; g Pharmaceutical Society of Nigeria, Lagos, Nigeria.; h HELP Logistics Ltd, Nairobi, Kenya.; i Medicines, Technologies, and Pharmaceutical Services Program, Management Sciences for Health, Nairobi, Kenya.; j National Malaria Elimination Programme, Federal Ministry of Health, Abuja, Nigeria.; k Directorate of Pharmaceutical Services, Freetown, Sierra Leone.

## Abstract

Efficient and secure supply chains are vital for effective health services worldwide. In low- and middle-income countries, the accessibility, affordability and availability of essential medicines, including antimicrobials, remain challenging. Ineffective supply chains often cause antimicrobial shortages, leading to inappropriate use of alternative agents and increasing the risk of antimicrobial resistance. Shortages, coupled with insecure supply chains, also encourage the infiltration of substandard and falsified medicines, leading to suboptimal treatment and further promoting antimicrobial resistance. Addressing antimicrobial supply-chain issues should be considered a key component of antimicrobial stewardship programmes. We have explored the link between medicine supply chains and antimicrobial use in seven focus countries: Kenya, Malawi, Nigeria, Sierra Leone, Uganda, United Republic of Tanzania and Zambia. We explored country medicine supply-system structures, national medicine supply-chain policy documents and global study reports. Our aim was to develop evidence-based strategies to enhance the effectiveness and efficiency of the medicine supply chains in supporting antimicrobial stewardship efforts. Better management of medical supply chains involves rational selection, quantification, forecasting, procurement, storage, distribution, use and stock management of antimicrobials. Important supply-chain considerations include pooled procurement networks to ensure consistent pricing of quality-assured antimicrobials, and improved resource utilization and information exchange among relevant stakeholders. We propose adaptable recommendations for integrating medicine supply chains as an essential part of antimicrobial stewardship programmes, with a call for action at the local, regional and national levels in low- and middle-income countries.

## Résumé

Partout dans le monde, les performances des services de santé dépendent de l'efficacité et de la sécurité des chaînes d'approvisionnement. Mais dans les pays à revenu faible et intermédiaire, l'accessibilité et la disponibilité des médicaments à prix abordable, y compris des antimicrobiens, représentent toujours un défi. L'inefficacité des chaînes d'approvisionnement entraîne souvent des pénuries d'antimicrobiens et, par conséquent, un recours à des alternatives inappropriées et une augmentation du risque de résistance aux antimicrobiens. Ces pénuries, alliées à des chaînes d'approvisionnement peu fiables, favorisent également l'introduction de médicaments falsifiés et de qualité inférieure, altérant l'efficacité du traitement et renforçant encore davantage la résistance aux antimicrobiens. Résoudre les problèmes liés aux chaînes d'approvisionnement en antimicrobiens devrait donc figurer parmi les priorités des programmes de gestion des antimicrobiens. Le présent document s'intéresse au lien entre les chaînes d'approvisionnement en médicaments et l'utilisation d'antimicrobiens dans sept pays cibles: le Kenya, le Malawi, le Nigeria, l'Ouganda, la République-Unie de Tanzanie, la Sierra Leone et la Zambie. Pour chacun de ces pays, nous avons examiné les structures du système d'approvisionnement en médicaments, les documents relatifs à la politique d'approvisionnement national et les rapports d'études globaux. Notre objectif consistait à développer des stratégies fondées sur des données factuelles, afin d'améliorer le fonctionnement et l'efficacité des chaînes d'approvisionnement en médicaments et de contribuer ainsi aux efforts de gestion des antimicrobiens. Une meilleure logistique requiert une certaine rationalité dans la sélection, la quantification, la planification, l'approvisionnement, le stockage, la distribution, l'utilisation et la gestion des stocks d'antimicrobiens. Dans ce contexte, plusieurs éléments sont importants tels que les réseaux d'achats groupés, qui assurent la stabilité des prix pour des antimicrobiens de qualité garantie, ou encore l'optimisation des ressources et l'échange d'informations entre les acteurs concernés. Nous formulons des recommandations ajustables en vue de rendre les chaînes d'approvisionnement en médicaments incontournables dans les programmes de gestion des antimicrobiens, avec un appel à agir à l'échelle locale, régionale et nationale dans les pays à revenu faible et intermédiaire.

## Resumen

Unas cadenas de suministro eficientes y seguras son vitales para la eficacia de los servicios sanitarios en todo el mundo. En los países de ingresos bajos y medios, la accesibilidad, asequibilidad y disponibilidad de los medicamentos esenciales, incluidos los antimicrobianos, sigue siendo un reto. Con frecuencia, las cadenas de suministro ineficaces provocan escasez de antimicrobianos, lo que conlleva un uso inadecuado de agentes alternativos y aumenta el riesgo de resistencia a los antimicrobianos. La escasez, sumada a la inseguridad de las cadenas de suministro, también favorece la infiltración de medicamentos de calidad inferior y adulterados, lo que conduce a un tratamiento subóptimo y fomenta aún más la resistencia a los antimicrobianos. Abordar los problemas de la cadena de suministro de antimicrobianos se debería considerar un componente clave de los programas de administración de antimicrobianos. Hemos explorado la relación entre las cadenas de suministro de medicamentos y el uso de antimicrobianos en siete países seleccionados: Kenia, Malawi, Nigeria, Sierra Leona, Uganda, República Unida de Tanzania y Zambia. Exploramos las estructuras de los sistemas de suministro de medicamentos de los países, los documentos de política nacional sobre la cadena de suministro de medicamentos y los informes de estudios globales. Nuestro objetivo era desarrollar estrategias basadas en evidencias para mejorar la eficacia y la eficiencia de las cadenas de suministro de medicamentos en apoyo de los esfuerzos de administración antimicrobiana. Una mejor gestión de las cadenas de suministro de medicamentos implica la selección racional, la cuantificación, la previsión, la adquisición, el almacenamiento, la distribución, el uso y la gestión de las existencias de antimicrobianos. Entre las consideraciones importantes sobre la cadena de suministro se incluyen las redes de adquisición mancomunada para garantizar precios coherentes de antimicrobianos de calidad garantizada y una mejor utilización de los recursos e intercambio de información entre las partes interesadas pertinentes. Proponemos recomendaciones adaptables para integrar las cadenas de suministro de medicamentos como parte esencial de los programas de administración de antimicrobianos, con una llamada a la acción a nivel local, regional y nacional en los países de ingresos bajos y medios.

## ملخص

تعد سلاسل الإمداد الفعالة والآمنة مطلبًا حيويًا لتقديم خدمات صحية فعالة في جميع أنحاء العالم. إن إمكانية الحصول على الأدوية الأساسية، بما يشمل مضادات الميكروبات، والقدرة على تحمل تكاليفها، فضلاً عن توافرها، إنما يمثل ذلك تحديًا في الدول ذات الدخل المنخفض والدخل المتوسط غالبًا ما تسبب سلاسل الإمداد غير الفعالة نقصًا في مضادات الميكروبات، مما يؤدي إلى الاستخدام غير المناسب للعوامل البديلة، وزيادة مخاطر مقاومة مضادات الميكروبات. كما أن حالات النقص المقترنة بسلاسل الإمداد غير الآمنة، تشجع على تسرب الأدوية غير القياسية والمغشوشة، مما يؤدي إلى علاج دون المستوى الأمثل، ويزيد من تعزيز مقاومة مضادات الميكروبات. إن التعامل مع قضايا سلسلة إمداد مضادات الميكروبات، يجب اعتبارها مكونًا رئيسيًا في برامج الإشراف على مضادات الميكروبات. لقد قمنا باستكشاف الرابط بين سلاسل إمداد الأدوية واستخدام مضادات الميكروبات في سبع دول للتركيز: كينيا وملاوي ونيجيريا وسيراليون وأوغندا وجمهورية تنزانيا المتحدة وزامبيا. استكشفنا هياكل أنظمة إمداد الأدوية في الدولة، ووثائق سياسة سلسلة الإمداد الوطنية للأدوية، وتقارير الدراسات العالمية. كان هدفنا هو تطوير استراتيجيات قائمة على الأدلة لتعزيز فعالية وكفاءة سلاسل الإمداد بالأدوية بهدف دعم جهود الإشراف على مضادات الميكروبات. تتضمن الإدارة الأفضل لسلاسل الإمداد الطبية: الاختيار العقلاني، والتقدير الكمي، والتوقع، والمشتريات، والتخزين، والتوزيع، والاستخدام، وإدارة مخزون مضادات الميكروبات. تشمل الاعتبارات المهمة لسلسلة الإمداد شبكات المشتريات المجمعة لضمان التسعير المتسق لمضادات الميكروبات مضمونة الجودة، وتحسين استخدام الموارد، وتبادل المعلومات بين أصحاب المصلحة المعنيين. نحن نقترح توصيات قابلة للتكيف لدمج سلاسل إمداد الأدوية كجزء أساسي من برامج الإشراف على مضادات الميكروبات، مع دعوة للعمل على المستويات المحلية والإقليمية والوطنية في الدول ذات الدخل المنخفض والدخل المتوسط.

## 摘要

高效而安全的供应链对于在全球范围内有效提供卫生服务至关重要。在中低收入国家，包括抗菌药物在内的基本药物的可及性、可负担性和可得性仍然具有挑战性。无效的供应链通常会导致抗菌药物短缺、替代药物的不当使用以及患者抵制使用抗菌药物的风险增加。短缺，再加上不安全的供应链，也助长了劣药和假药的渗透，导致治疗效果不佳，从而进一步促使患者抵制使用抗菌药物。解决抗菌药物供应链问题应被视为抗菌药物管理项目的一个关键组成部分。我们在肯尼亚、马拉维、尼日利亚、塞拉利昂、坦桑尼亚联合共和国、乌干达和和赞比亚这七个重点国家探索了药品供应链与抗菌药物使用率之间的联系。我们研究了国家药品供应系统结构、国家药品供应链政策文件和全球研究报告。我们的目标是制定循证策略，以提高药品供应链在支持抗菌药物管理工作方面的有效性和效率。好的医疗供应链管理涉及抗菌药物的合理选择、量化、预测、采购、储存、分配、使用和库存管理。重要的供应链考虑因素包括集中采购网络，以确保抗菌药物的质量有保证且价格一致，提高资源利用率以及加强相关利益攸关方之间的信息交流。我们提出了适用性强的建议，将药品供应链整合为抗菌药物管理项目的重要组成部分，并呼吁中低收入国家在地方、区域和国家层面采取行动。

## Резюме

Эффективные и надежные цепочки поставок жизненно важны для результативного оказания услуг здравоохранения по всему миру. В странах с низким и средним уровнем доходов по-прежнему существует проблема доступности, в том числе ценовой, и наличия основных лекарственных средств, включая антимикробные препараты. Неэффективные цепочки поставок часто вызывают дефицит антимикробных препаратов, что приводит к нецелесообразному использованию альтернативных средств и повышает риск развития устойчивости к антимикробным препаратам. Дефицит в сочетании с ненадежными цепочками поставок также способствует распространению некачественных и контрафактных лекарственных средств, что приводит к неоптимальному лечению и еще больше способствует развитию устойчивости к антимикробным препаратам. Решение вопросов, связанных с цепочкой поставок антимикробных препаратов, следует рассматривать как ключевой компонент программ рационального использования антимикробных препаратов. Изучалась связь между цепочками поставок лекарственных средств и использованием антимикробных препаратов в семи целевых странах: Кении, Малави, Нигерии, Объединенной Республике Танзания и Замбии, Сьерра-Леоне, Уганде. Также были изучены структуры систем поставок лекарственных средств в странах, документы по национальной политике в области поставок лекарственных средств и отчеты о глобальных исследованиях. Цель заключалась в разработке стратегий, основанных на фактических данных, для повышения эффективности и результативности цепочек поставок лекарственных средств в поддержке усилий по рациональному использованию антимикробных препаратов. Более эффективное управление цепочками поставок лекарственных средств предполагает рациональный выбор, количественную оценку, прогнозирование, закупку, хранение, распределение, использование запасов антимикробных препаратов, а также управление ими. К важным аспектам цепочки поставок относятся объединенные сети закупок для обеспечения согласованного ценообразования на качественные антимикробные препараты и улучшения использования ресурсов и обмена информацией между соответствующими заинтересованными сторонами. Предлагаются адаптируемые рекомендации по интеграции цепочек поставок лекарственных средств в качестве важной части программ по рациональному использованию антимикробных препаратов с призывом к действиям на местном, региональном и национальном уровнях в странах с низким и средним уровнем доходов.

## Introduction

Antimicrobial resistance disproportionately affects low- and middle-income countries.^1^ An often-missing component of governance surrounding antimicrobial resistance in many countries is medicine procurement and inventory control. Antimicrobial stewardship programmes, which aim to optimize the use of antimicrobials, may not succeed unless medicine procurement is effective, efficient and secure, ensuring the timely availability of appropriate antimicrobial products.^2^

Health supply chains are the processes and operations required to get products and medicines from manufacturers to health-care staff and patients at the right place, price, time, cost, quality, efficacy and quantity. Strong and secure supply chains save lives. They are the fundamental building blocks of national welfare systems and are necessary for the achievement of the sustainable development goals. The coronavirus disease 2019 (COVID-19) pandemic drew attention to the importance of resilient supply chains in national emergency response efforts.^3^

A continuing challenge in low- and middle-income countries is inadequate access to essential medicines and medical supplies, including antimicrobial medicines.^4^ High-income countries also report challenges with medicine supplies, but these countries frequently have advanced reporting systems and, in many cases, easier access to substitute medicines.^5^ Despite this access, a survey conducted by the European Association of Hospital Pharmacists revealed that 86% of hospital pharmacists across 30 countries (463 out of 537 respondents), reported that medicine shortages were a current problem in their hospitals, with the majority reporting that this problem occurred on a daily or weekly basis.^6^ Antimicrobial shortages hinder timely access to effective therapies and can be drivers for the emergence of antimicrobial resistance and excess mortality, particularly in low- and middle-income countries.^7^ Infiltration of substandard and falsified medicines into the medicine supply system can occur via unauthorized suppliers or due to inadequate enforcement of regulations. Commodity insecurity in lower-resource settings therefore also has an impact on the quality and effectiveness of antimicrobials.

There is limited published information specifically on the supply chains for antimicrobials and access to these medicines in low- and middle-income countries. In this article we explore the impact of medicine supply chains on antimicrobial use, focusing on seven countries of the Commonwealth Partnerships for Antimicrobial Stewardship programme. We issue a call to action on incorporating supply-chain considerations into antimicrobial stewardship policies and processes.

## Countries overview

Table 1 provides an overview of the medicine supply chains of the seven focus countries: Kenya, Malawi, Nigeria, Sierra Leone, Uganda, United Republic of Tanzania and Zambia. We highlight the similarities and differences among them in the type of procurement agency; the public service agency responsible for the procurement, warehousing and distribution of drugs and medical supplies; and the availability of a national essential medicine list for public institutions. We based our overview on a purposeful search of each country’s medicine supply-system structures, national medicine supply-chain policy documents, global study reports and a rapid literature search of PubMed^®^ articles published between 2015 and 2021. 

**Table 1:** Overview of the medicine supply chains in seven African countries

Country	Government procurement agency	Nongovernmental organization procurement agency	Private sector procurement agency	Supply-chain system	Centralized supply chain	Decentralized supply chain	Essential medicines list and website link	AWaRe antibiotic categories recommended in essential medicines list
Kenya	Yes	Yes	Yes	Three supply-chain systems	Yes	No	Kenya Essential Medicines List, 2019 https://www.health.go.ke/wp-content/uploads/2020/07/Kenya-Essential-Medicines-List-2019.pdf	Yes
Malawi	Yes	Yes	Yes	Single agency	Yes	No	Malawi Standard Treatment Guidelines 5th edition, 2015 https://eYestranet.who.int/ncdccs/Data/MWI_D1_Malawi-Standard-Treatment-Guidelines-Essential-Medicines-List-2015.pdf	No
Nigeria	Yes	Yes	Yes	National body devolved to states	No	Yes	Nigeria Essential Medicine List, 2020 7th edition https://www.health.gov.ng/doc/Nigeria-Essential-Medicine-List-2020.pdf	No
Sierra Leone	Yes	Yes	Yes	Single agency	Yes	No	Sierra Leone Basic Packaging of Essential Health Service, 2015–2020 https://mohs2017.files.wordpress.com/2017/06/gosl_2015_basic-package-of-essential-health-services-2015-2020.pdf	No
Uganda	Yes	Yes	Yes	Single agency	Yes	No	Essential Medicines and Health Supplies List for Uganda, 2016 https://www.health.go.ug/cause/essential-medicines-and-health-supplies-list-2016/	No
United Republic of Tanzania	Yes	Yes	Yes	Single agency	Yes	No	Standard Treatment Guidelines & National Essential Medicine List United Republic of Tanzania Mainland, 2017 https://hssrc.tamisemi.go.tz/storage/app/uploads/public/5ab/e9b/b21/5abe9bb216267130384889.pdf	Yes
Zambia	Yes	Yes	Yes	Three-tier system	Yes	No	Zambia Essential Medicines List, 2020 https://www.moh.gov.zm/?wpfb_dl=39	Yes

AWaRe: World Health Organization Access, Watch, Reserve classification of antibiotics.^8^

All seven countries obtain, fund and distribute essential medicines through a combination of health ministries, nongovernment organizations and the private sector. The existing systems are classified as tier systems, single agencies or devolved. Except for Nigeria, every country has a centralized supply chain. All seven countries have existing national essential medicine lists. Kenya, United Republic of Tanzania and Zambia have WHO AWaRe (Access, Watch, Reserve) categories of antimicrobials recommended in their national essential medicine lists. All seven countries face challenges in the management of health products and technologies that are familiar to many high-income countries. Challenges reported in medicine supply chains include: inadequate quality assurance; weak regulation; poor enforcement of laws; unavailable and unaffordable medicines; transport and storage infrastructure; poor information and logistics management; inadequate human resources; and inadequate financing.^9^^–^^15^

### Kenya

Local supply chains for medicines and medical supplies in Kenya are dependent on three supply chains within the health sector: the health ministry; non-profit procurement agencies; and private distributors and wholesalers.^16^ The health ministry provides stewardship over the supply chain for health products and technologies as guided by the Health Policy 2014–2030.^17^ The health ministry, with support from implementation partners, has rolled out several capacity-building initiatives. The initiatives focus on improving the accuracy of medication quantification and forecasting; optimizing commodity procurement and storage practices; and enhancing the effectiveness of the logistics management information system to better track and manage the flow of medicines and supplies.^9^

Shortages of essential medicines in Kenya^10^ have resulted in substitution of publicly funded first-line treatments with second-line drugs, leading to increased out-of-pocket expenditure on medicines by patients using the private health sector. Higher drug costs can lead to patients purchasing substandard and falsified medicines through unlicensed and illegal pharmacies or unregulated websites. To help address the situation, hospitals in Kenya conduct ABC (always, better, and control) and VEN (vital, essential, and non-essential) analysis of their medicine use. Research into current patient and epidemiological patterns helps Kenya to understand the specific health needs of its population and inform its medication selection and prescribing practices.^18^^–^^20^

### Malawi

The supply chain for essential medicines in Malawi follows a rigorous process that ensures compliance with the Public Procurement Act (2003) and the Public Procurement Guidelines (2004). The Central Medical Stores Trust sources medicines and other health commodities in accordance with the policies and guidelines of the Director of Public Procurement. Essential medicines are distributed from a central warehouse to three regional medical stores which supply public health facilities.^11^

Malawi has other parallel supply-chain systems for essential medicines, especially relating to disease programmes such as tuberculosis, malaria and reproductive health. Chronic stockouts of medicines at the central medical stores and in health facilities remain a challenge.^11^

### Nigeria

There are two key agencies in Nigeria involved in medicine supplies: the National Agency for Food and Drug Administration and Control; and the Pharmacists Council of Nigeria. The pharmaceutical services division within the Nigerian food and drug authority is part of the health ministry and regulates and monitors the pharmaceutical supply chain, supporting access to quality medicines in Nigeria.^21^

Nigeria supplies agencies have faced difficulties with selection of medicines and vaccines, procurement and distribution of medicines, inventory management and storage infrastructure.^12^ These challenges have led to stock-outs of essential medicines.

### Sierra Leone

The National Medical Supplies Agency of Sierra Leone, formerly the Directorate of Drugs and Medical Supplies, undertakes the transparent and cost–effective procurement, warehousing and distribution of drugs and medical supplies on behalf of all public institutions throughout the country.^7^^,^^22^ Several additional parallel supply chains exist, including those for specific programmatic areas such as human immunodeficiency virus infection, tuberculosis, malaria, nutrition and neglected tropical diseases. This fragmentation dilutes resources and capacity, duplicates activities and creates administrative inefficiencies.^23^

The impact of the COVID-19 pandemic on the local pharmaceutical industry resulted in severe drug shortages and medicine insecurity in the country due to the restrictions imposed on cross-border imports.^24^ Sierra Leone depends solely on the import of pharmaceutical commodities, which creates an opportunity for substandard and falsified medicines to enter the legitimate medicine supply chain.^24^ Sierra Leone’s National Medical Supplies Agency also partners with external agencies to develop efficient procurement, distribution and inventory systems.^13^

### Uganda

The management of essential medicines and health supplies in Uganda is guided by the Essential Medicines and Health Supplies Management Manual of 2018. The National Medical Stores provide the public health-care facilities with the core antibiotics.^25^

As in many other low- and middle-income countries, Uganda faces challenges with enforcement of regulations, creating multiple nodes of entry for medicines into the market that may accelerate the proliferation of substandard and falsified products.^26^ Challenges to achieving sustainable medicine use in Uganda include poor forecasting and planning of medical supplies, overly bureaucratic procurement guidelines, delays in shipping of medicines and poor supply-chain infrastructure.^27^ As a result, patients, health-care providers and regulatory agencies have reported fragmented access to essential medicines, inappropriate use of medicines, poor quality and inappropriate disposal of medicines and inadequate information. 

### United Republic of Tanzania

The United Republic of Tanzania has a centralized procurement system for medicines. The Medical Stores Department, under the Public Procurement Act 2004 and Regulations 2005, is empowered to advertise, receive, evaluate and award successful bidders for government contracts. The price and quality of the product are given equal consideration when awarding the tender.^28^

The country has a higher use of antimicrobials compared with many other countries worldwide. Contributing factors include a relatively higher burden of infectious diseases; limited diagnostic services in health facilities; widespread availability of antibiotics without prescription; and the unexplained use of certain antibiotics in the animal health sector.^14^

### Zambia

Zambia has a three-tier public sector distribution system for essential drugs. The primary distribution of drugs and other health commodities is managed by the Zambia Medicines and Medical Supplies Agency, formerly Medical Stores Limited. The secondary distribution of commodities falls under the responsibility of district health management teams reporting to the health ministry. The national pharmacovigilance unit under Zambia’s Medicines Regulatory Authority leads and coordinates the pharmacovigilance or drug safety monitoring programme in the country.^29^

According to Zambia’s national health strategic plan, the key planning issues for the medicines, vaccines and medical supplies sector are weak coordination mechanisms and accountability in supply-chain management, and an inadequate quality management system for data in the supply chain.^15^

## Missing links

Challenges within antimicrobial supply chains worldwide, including in low- and middle-income countries, make forecasting and mitigating antimicrobial shortages difficult.^7^ Research findings suggest that a combination of factors create barriers to access that prevent millions of people from receiving the antibiotics they need. These factors include weak, underfunded health systems, failure to reimburse suppliers, unreliable supply chains and high out-of-pocket costs.^30^^,^^31^ Affordability of medicines has also affected decisions on antimicrobial use in Kenya and northern United Republic of Tanzania.^32^ Shortages and stock-outs can be prevented by strengthening the supply chain for medicines across all stages, from active pharmaceutical ingredients to finished products, including ensuring robust quality of pharmaceutical ingredients and all raw materials.^33^ The fragility of supply chains for medicines was highlighted during the COVID-19 pandemic. Closures of country borders, spikes in demand for medicines and supply interruptions overwhelmed governments, pharmaceutical companies and health systems.^34^ Such conditions not only affect access to essential medicines, but also present opportunities for substandard and falsified medicinal products to infiltrate the supply chain. These medicines enter through various channels, such as counterfeiters, unauthorized distributors or other groups who may take advantage of weaknesses in the supply chain.^35^ For example, the availability of cardiovascular disease medicines is reported to be low in rural western Kenya, fuelling the rise of substandard and falsified medicines in this category.^36^

Antimicrobials, especially antimalarials and antiretrovirals, are the most commonly falsified medicinal products worldwide.^37^ Suboptimal antimicrobial doses caused by the use of poor quality or counterfeit products accelerate the development of antimicrobial resistance. In many low- and middle-income countries, patients can purchase antibiotics without prescription. Studies show that over-the-counter antibiotics may be used as incomplete regimens or to treat non-bacterial illnesses.^32^ The problem is exacerbated by shortages and frequent stock-outs of essential medicines, including antimicrobials. Supply-chain security is therefore an important factor in antimicrobial resistance.^37^ Poor inventory management, limited logistics management information systems, inadequate storage systems and weak quality control can increase the risk of substandard and falsified antimicrobials entering supply chains. In many low- and middle-income settings, ineffective drug distribution systems increase the chances of expired drugs entering the pharmaceutical supply chain, creating waste, and compromising quality and health outcomes relating to the safe and effective use of medicines.^12^^,^^38^ Furthermore, inadequate access to basic health services may lead patients to obtain supplies from less-regulated sources, such as informal markets, increasing their risk of acquiring substandard and falsified medicines.^35^

The main functions of countries’ national medicines regulatory authorities include: (i) control of pharmaceutical products through registration and post-marketing surveillance; (ii) control of activities by licensing and inspection of manufacturers, importers, exporters, wholesalers, distributors, pharmacies and retail outlets; (iii) control of clinical trials; and (iv) control of promotion of pharmaceuticals. However, in 2010 the World Health Organization (WHO) estimated that 34 out of 38 national medicines regulatory authorities in sub-Saharan Africa were not enforcing basic regulatory functions.^39^ The task of the regulatory authorities is complicated by the complexity of the international supply chains, whereby intermediate traders and brokers operate along transnational routes outside stringent regulatory supervision. Furthermore, the major donors to medical programmes do not always apply consistent quality criteria for the selection of the medicines that they fund. International and national procurement agencies play an important role in defining the quality of medicines available at country level.^39^

Drug shortages, combined with inappropriate prescribing and use of antimicrobials, accelerate the development of antimicrobial resistance and hence reductions in antimicrobial effectiveness.^31^ Access to medicines, including antimicrobials, is hampered by supply-chain bottlenecks such as inadequate medicine selection, use, procurement and inventory management.^40^ In Malawi, supply-chain bottlenecks have been reported to prevent community health workers from accessing essential medicines for vulnerable children in poor and rural areas.^40^ Health-care workers can improve supply chains by implementing better supply-chain management.^41^

Pharmacists have an important role to play.^41^ Storage and distribution are essential components of medicine supply chains. Pharmacists can help to design and implement processes to strengthen supply-chain security by regulating appropriate medicine distribution and reporting of substandard and falsified medicinal products. Various assessments conducted in Zambian health facilities identified secondary distribution, from district stores to health facilities as the main bottleneck in the distribution system.^29^ Inappropriate storage conditions during transport, such as exposure to high temperatures or moisture, may affect the stability of antimicrobials and lead to suboptimal doses of the drugs, further fuelling antimicrobial resistance.^42^ These processes are often managed by pharmacists, who can advise on and monitor storage conditions and hence play a role in antimicrobial stewardship. Countries can also reduce overuse of antimicrobials by ensuring adequate vaccination coverage for relevant diseases.^43^^,^^44^ A secure and effective vaccine supply chain (including adequate cold-chain maintenance where appropriate) is fundamental to achieving vaccination goals.

## Looking ahead

Addressing supply-chain challenges requires consistent efforts at the global, national and regional levels, building on existing partnerships and legislation. We need to address ways to pool the procurement of medicines, improve the forecasting of supply needs and increase the commitment to improving processes and resources. Measures could include appropriate storage conditions, implementing efficient transportation and distribution systems, and maintaining reliable quality control measures throughout the supply chain. Sustainability of medicine supply chains remains an elusive goal that is yet to be integrated into discussions.^45^ There is evidence that a multi-pronged approach is effective to improve the supply chain of medicines in hospitals. Such an approach includes proactive management of the supply chain, accurate forecasting of supply needs and the implementation of therapeutic interchange policies to ensure the availability of substitute medications when needed.^30^^,^^46^ Ensuring access to medicines of a suitable quality is an important aspect of effective health systems. Proper inventory management regulated by pharmacists can result in a consistent supply of the medicines and vaccines necessary for the successful implementation of antimicrobial stewardship programmes. Building inventory management principles into antimicrobial stewardship will support countries to acquire medications that meet good manufacturing practices and are free from adulteration and tampering.

We can improve antimicrobial stewardship and strengthen the knowledge and evidence base of factors contributing to antimicrobial resistance through ongoing surveillance and research.^2^ Evidence-based research can inform effective policy guidance to improve many aspects of the supply and use of antimicrobials, including supply-chain management. Furthermore, implementation of WHO essential medicine policies is important for reducing antimicrobial misuse in low- and middle-income countries.^47^ Such efforts could improve access to medicines by increasing stock availability and decreasing the prevalence of substandard and falsified antimicrobials.^48^

It is important to ensure that, as far as possible, appropriate Access and Watch antibiotics are available for the most common infections within hospitals across Africa. The *WHO AWaRe (Access, Watch, Reserve) antibiotic book* offers clear and evidence-based recommendations on the appropriate antibiotic choice, dosage, administration route and treatment duration for over 30 prevalent clinical infections in both primary health care and hospital settings. The book’s information aligns with the antibiotic recommendations outlined in the *WHO Model list of essential medicines* and *WHO Model list of essential medicines for children*, as well as the WHO AWaRe antibiotic classification.^8^ The *African antibiotic treatment guidelines for common bacterial infections and syndromes* also provide care workers with expert recommendations for antimicrobial selection, dosage and duration of treatment for common bacterial infections and syndromes among paediatric and adult patient populations in Africa.^49^ To ensure the continuity of medication supply, there is an increasing demand for and evidence to support the use of digital technology as a tool to monitor stock levels, which provides accurate and real-time feedback.^42^

Post-marketing surveillance and pharmacovigilance is needed to detect substandard and falsified medicinal products. Efforts are also required to improve medicine quality by strengthening the medicine distribution chain; ensuring the use of quality-assured drugs through improved pharmaceutical governance; and strengthening the technical capacity of regulatory laboratories, particularly in poor and rural communities.^48^ Approaches such as the Lomé Initiative – a binding agreement to criminalize the trafficking of substandard and falsified medical products in some African countries – may also increase access to quality medicines.^50^

We propose that the basic principles of medicine supply-chain systems should be included in capacity-building for antimicrobial stewardship teams and health-care personnel at local, national and global levels. The managers of procurement systems within a country are responsible for ensuring the continuous availability of essential quality-assured medicines at an affordable price. Implementing monitoring and evaluation systems for antimicrobial resistance and antimicrobial use as part of national action plans could contribute to the use of quality-assured medicines, and reduce the distribution of substandard and falsified medical products.^51^

## Proposed recommendations

In the absence of stringent national and international regulatory oversight, ensuring the quality of medicines becomes a choice rather than a duty. As a result, there is a risk that poor-quality medicines will enter the supply chain, causing harm to individuals and public health.^46^ We propose that medicine supply chains be considered an integral part of antimicrobial stewardship, and we issue a call to action to address the issue at the local, regional and national levels in low- and middle-income countries (Box 1). We present adaptable recommendations for establishing antimicrobial stewardship programmes that include management of medical supply chains at local levels; streamlining and strengthening national supply-chain systems; and improved resource utilization and information exchange among relevant stakeholders. These actions will ensure that adequate supplies of quality antimicrobials are available, allowing health professionals to select appropriate treatments and contributing to safe and effective use of antimicrobials.

Box 1 **Proposed adaptable recommendations for local, regional and national organizations in low- and middle-income countries**

***Local organizations***

- Optimize appropriate antimicrobial use through pre-service and in-service training for health-care workers on effective medicine procurement, inventory control and distribution management.
- Designate local resource mobilization as a priority area in health ministries to build and sustain the medicine supply-chain systems.
- Engage the right human workforce into the local supply chains to ensure reliability of the systems (supply planning experts, procurement professionals, commodity specialists, pharmacists, pharmaceutical technologists, quality assurance staff, security personnel, storage professionals and logistics experts).
- Establish or empower medicine and drug and therapeutics committees to act as an oversight mechanism in health-care facilities.
- Establish antimicrobial stewardship programmes that include supply-chain management as an essential component, to ensure rational antimicrobial selection, quantification and forecasting, procurement, storage, distribution, use and stock management of antimicrobials.

***Regional organizations***

- Streamline and strengthen national medicine supply-chain systems.
- Use pooled procurement networks, to ensure consistent pricing of quality-assured antimicrobials and to improve resource utilization and information exchange between organizations.
- Harmonize drug master files and specifications required for antimicrobial resistance surveillance.
- Standardize pre-qualified supply mechanisms, including those from manufacturers, to ensure constant availability of antimicrobials.
- Increase the number of well-designed studies into medicine supply-chain management to identify gaps and challenges in low- and middle-income countries.
- Increase quality assurance programmes that conduct routine inspections of manufacturers, suppliers and warehouses to ensure medicines meet international quality standards.

***National organizations***

- Consider approaches such as the Lomé Initiative to criminalize the trafficking of falsified medical products.
- Increase regulatory capacity and reporting mechanisms to detect and tackle substandard and falsified medical products.
- Strengthen national medicines regulatory agencies and harmonize legislation, taking a collaborative approach through the African Medicines Agency.
- Increase local production of antimicrobials to strengthen supply-chain resilience and reduce the risk of infiltration of substandard and falsified antimicrobials.
- Encourage more national policies and government regulatory frameworks for the essential infrastructure, such as national warehousing, distribution and transport of quality-assured health products.
- Incorporate analysis of bottlenecks into national supply chains to identify underlying causes and human behaviours that influence procurement agencies and practices.
- Integrate digital health systems interventions, with the implementation and scale-up of logistics management information system software, to improve the forecasting, monitoring, evaluation, tracking and planning of medicine supply chains.
- Ensure that appropriate Access and Watch antibiotics are available for key diseases relevant to the national context and the World Health Organization* Model list of essential medicines*.
- Set up consumption surveillance systems for antimicrobials to ensure rational antimicrobial selection and use, particularly lower down the supply chain.
- Develop and implement antimicrobial national action plans to include supply-chain management.
- Ensure antimicrobial stewardship initiatives address over-prescribing and inappropriate prescribing; improve patient adherence to treatment regimens; and address potential environmental contamination, for example through inappropriate disposal of antimicrobials.
- Update mechanisms for the review and reporting of substandard and falsified medicines, providing a user-friendly and safe environment to encourage reporting and facilitate feedback.
